# Workability and Flexural Properties of Fibre-Reinforced Geopolymer Using Different Mono and Hybrid Fibres

**DOI:** 10.3390/ma14164447

**Published:** 2021-08-08

**Authors:** Jacob Junior, Ashish Kumer Saha, Prabir Kumar Sarker, Alokesh Pramanik

**Affiliations:** School of Civil and Mechanical Engineering, Curtin University, Perth, WA 6102, Australia; Jacob.junior@kier.co.uk (J.J.); p.sarker@curtin.edu.au (P.K.S.); Alokesh.pramanik@curtin.edu.au (A.P.)

**Keywords:** deflection hardening, fibre-reinforced geopolymer mortar, flexural strength, residual strength factor, toughness index

## Abstract

The effects of mono (single type) and hybrid (mixed types) fibres on the workability, compressive strength, flexural strength, and toughness parameters of fly ash geopolymer mortar were studied. The ratio of sand to geopolymer paste of the mortar was 2.75. It was found that workability of mortar decreased more with the use of PP fibres due to its higher dispersion into individual filaments in geopolymer mortar compared to the bundled ARG and PVA fibres. Compressive strength increased by 14% for using 1% steel with 0.5% PP fibres compared to that of the control mixture, which was 48 MPa. However, 25 to 30% decrease of compressive strength was observed in the mortars using the low-modulus fibres. Generally, flexural strength followed the trend of compressive strength. Deflection hardening behaviours in terms of the ASTM C1609 toughness indices, namely I5, I10 and I20 were exhibited by the mortars using 1% steel mono fibres, 0.5% ARG with 0.5% steel and 1% PVA with 0.5% steel hybrid fibres. The toughness indices and residual strength factors of the mortars using the other mono or hybrid fibres at 1 or 1.5% dosage were relatively low. Therefore, multiple cracking and deflection hardening behaviours could be achieved in fly ash geopolymer mortars of high sand to binder ratio by using steel fibres in mono or hybrid forms with ARG and PVA fibres.

## 1. Introduction

Demand for construction materials is increasing with the increase of global population. The traditional binder for concrete is Portland cement, which is a significant contributor to the global carbon dioxide emission. Various attempts have been made to reduce the carbon emission from cement production, including the well-known partial replacement of cement by industrial slags and fly ash [1]. However, low early-age strength caused by the partial cement replacement using low-calcium fly ash is considered a drawback for many in-situ applications [2]. The development of geopolymers as a low-emission alternative binder has attracted significant interest since geopolymers can be manufactured using industrial by-products such as fly ash and ground granulated blast furnace slag (GGBFS) without using cement. Geopolymers are inorganic polymers where an aluminosilicate source material is commonly reacted with an alkali to produce the binder. Geopolymer mixtures can be tailored to achieve properties comparable to those of ordinary Portland cement (OPC) binders [3]. In a recent study, Bewa et al. [4] showed that geopolymers based on fired clay brick powder and an acid solution could develop high strength after room temperature curing. It was shown that higher amounts of amorphous silica and nanocrystalline hematite contents could accelerate the strength development. A study has shown that application of geopolymer can reduce greenhouse gas emissions by up to 60% over OPC [5]. The common industrial by-products used for geopolymers are fly ash and GGBFS since they are rich in reactive aluminosilicate. The commonly used activator liquids are sodium or potassium-based alkalis.

Low-calcium fly-ash based geopolymer usually hardens slowly at ambient temperature. This results in slow setting and low early age strengths [6] which also affects the microstructural development [7]. However, the setting and hardening can be accelerated by using a small proportion of a calcium-bearing material such as GGBFS [8] and by increasing the fineness of the fly ash [9].

Like the traditional OPC binder, geopolymer is also brittle in nature. Since brittle materials have no significant post-cracking ductility, they experience sudden failure when a fracture is initiated. Fibre reinforcements are used to improve composites’ performance under load, where the fibres transfer the tensile force across cracks and create a more homogenous composite in regard to its tensile properties [10]. Different types of fibres are used in a composite in order to control cracks and increase the fracture toughness by bridging both micro and macro cracks in the brittle matrix. Thus, fibres can improve the flexural strength, energy absorption and toughness of composites to various extents depending on the type and proportions of the fibre used. The post-cracking behaviour of a composite is defined by toughness parameters calculated using the area under the load-deflection curve [11]. Fibres can be used in mono or hybrid forms. The use of hybrid fibres utilises the benefits of different types of fibres in a composite by improvement of different properties.

Short fibres are usually found to be beneficial when used with paste or mortar. Fibres can be categorised into two groups: micro and macro short fibres. The term micro fibre is used where the fibre is less than 12.7 mm in length, and macro fibre for lengths greater than 12.7 mm [12]. Short fibres are randomly distributed in the mixture and therefore provide extra strength in all directions of the composite. It also means that there is more surface area, as the diameters of the short fibres are in the micro metres range. In other words, there are a greater number of fibres in the composite than that using long fibres [13]. As shown by Ahmed and Mihashi [14], short fibres exhibit a higher ultimate load due to their small size and higher interfacial area, leading to an increase in the maximum load to be transferred by the fibres. It was shown that hybridisation of different types of fibres can results in great improvements of the properties of composites [15], creating better composites for real-life applications. However, extensive studies are required to determine the most effective and efficient hybrid combinations of fibres with a specific binder.

Several recent studies have investigated the effects of fibres on the behaviour of geopolymer composites. Shaikh [16] used steel and polyvinyl alcohol (PVA) fibres in mono and hybrid combinations in geopolymer composites with sand to binder ratios of 0.5 and 0.75. It was shown that 2% volume fraction of fibres provided deflection hardening and multiple cracking of geopolymer composites. Ohno and Li [17] showed the high tensile ductility of fly ash geopolymer composites using PVA-ST fibres, and thus established the feasibility of developing strain-hardening ductile geopolymer composites. Bhutta et al. [18] studied the flexural behaviour of geopolymer composites using three different types of macro steel fibres with relatively high aspect ratio. It was shown that compressive strength, splitting tensile strength and flexural toughness significantly improved by the macro fibres, with the end-deformed fibres being more effective than the straight and length-deformed types. Sanjayan et al. [19] studied the fracture toughness of geopolymer pastes reinforced with up to 5% volume of steel fibres of 30 mm length and proposed a model to predict the fracture toughness by using finite element analysis.

Since geopolymers use viscous alkaline chemicals instead of water, addition of fibres in a geopolymer matrix may pose severe challenges in terms of workability of the fresh mixture. Though an increased percentage of fibre content may improve the post-cracking performance, a low workability may have negative effects on the setting behaviour, distribution of fibres and mechanical properties of the hardened composite. An adequate workability of the mixture is essential for proper mixing with uniform distribution of fibres, adequate setting time and proper compaction. Also, most previous studies on fibre- reinforced geopolymer composites used no sand or low sand to binder (fly ash) ratios such as 0.5. Geopolymer mixtures with lower binder contents would need less chemicals and hence significantly improve the economy and sustainability of geopolymer mixtures. Therefore, this study investigated the workability and mechanical properties of fibre-reinforced geopolymer mortars (FRGM) using a high sand to binder ratio such as 2.75 and reinforced with different types of mono and hybrid fibres. Workability was determined by flow test of fresh FRGM, and the mechanical properties were determined by compressive strength, flexural strength and toughness parameters of hardened FRGM specimens. The types of fibres used in this study were steel (S), alkali-resistant glass (ARG), polyvinyl alcohol (PVA) and polypropylene (PP) fibres. Effects of the mono and hybrid combinations of the fibres on workability and mechanical properties of FRGM were compared using the test results.

## 2. Materials and Methods

### 2.1. Material Properties and Mix Proportions

A class F fly ash was used as the primary aluminosilicate source to produce geopolymer. The silicon dioxide, aluminium oxide, iron oxide and calcium oxide contents of the fly ash were 53.7, 27.2, 11.7 and 1.9%, respectively. The mean particle size of fly ash and sand were 14 microns and 1.18 mm, respectively. A combination of 10 M sodium hydroxide solution and commercially available sodium silicate solution was used as the alkaline liquid. The sodium silicate consisted of 58.5, 30 and 11.5% of water, SiO_2_ and Na_2_O, respectively. The geopolymer mortar mix proportions are given in Table 1. The liquid to fly ash ratio was 0.6 and the mass ratio of sodium silicate to sodium hydroxide solutions was 2.5. A naphthalene-based superplasticiser was used to enhance workability of the fresh mixtures. 

**Figure 1:**
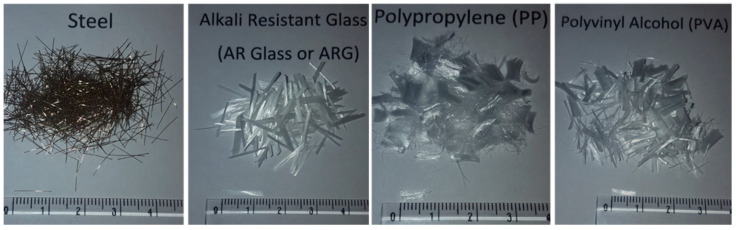
Physical appearance of the fibres.

The physical and mechanical properties of the fibres are given in Table 2. The lengths of the fibres varied from 6 to 13 mm. The aspect ratios were 83 for steel, 720 for ARG, 210 for PVA and 188 for PP fibre. The steel fibres had the highest and PP fibres had the lowest tensile strength and elastic modulus among all the fibres. The physical appearances of the fibres are shown in Figure 1. The ARG, PP and PVA fibres were chopped strands and the steel fibres were single needles. Some chopped strands may break down into individual filaments during mixing while some others may remain as bundles of filaments.

A control mortar mixture without any fibres and nine other mixtures were prepared using mono and hybrid combinations of the fibres. The fibre contents were 1 and 1.5% of the total volume of mortar. These fibre contents were selected considering the workability of the mixtures as higher fibre contents showed stiffer mixtures in the initial trials. Since steel fibres can be more prone to corrosion, the other types of fibres were used in larger proportions in the hybrid combinations. The mortar mixtures are designated by letters indicating the type of fibre and numbers indicating the volume percentage of the fibre. For example, the mixture containing 1% PVA fibre and 0.5% steel fibre is designated by PVA1.0-S0.5. The mix codes are listed in Table 3.

### 2.2. Mixing, Casting and Test Methods

The saturated surface dry (SSD) sand and fly ash were first mixed dry and then the alkaline liquid was added as the mixing continued. The fibres were then added slowly and the mixing continued until a uniform mixture of all the ingredients was achieved. Slump and flow tests of the fresh mixture was conducted as per ASTM C1437 [20] in order to determine the workability of FRGM. Then, the fresh mortar mixture was poured into 300 × 75 × 20 mm and 50 × 50 × 50 mm moulds for flexural test and compressive strength test, respectively. The size of the flexural test specimens was adopted from previous studies on the flexural behaviour of short fibre-reinforced geopolymer composites [16,21]. Compaction of the specimens was achieved by casting them on a vibrating table. After casting and finishing, the specimens were cured for 24 h at 70 °C. The samples were then demoulded and left in ambient condition until testing at the age 7 days.

The cube specimens were tested for compressive strength and the beam specimens were tested for flexural behaviour. A third-point loading test was performed using a clear span of 240 mm in deflection control mode to determine the flexural properties. The flexural test was conducted as per ASTM C1609 [22], with a deflection control mode of 0.1 mm/min until failure. Five identical specimens were tested for each test and the mean values of the results are reported in the following sections. The mid span deflection and corresponding load were recorded using LVDTs and an electronic data logger.

## 3. Results and Discussion

### 3.1. Workability

**Figure 2:**
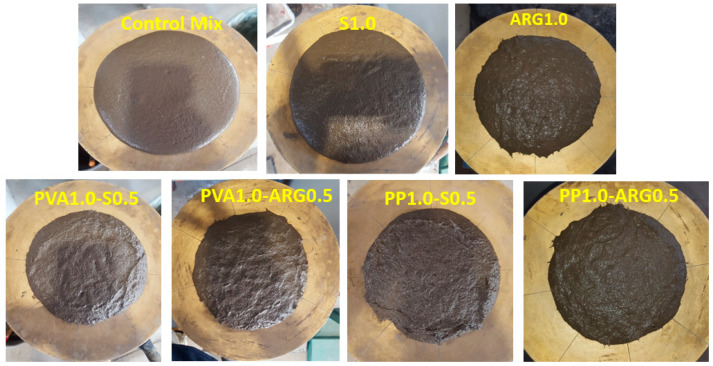
Flow of typical mortar mixtures using different fibres.

**Figure 3:**
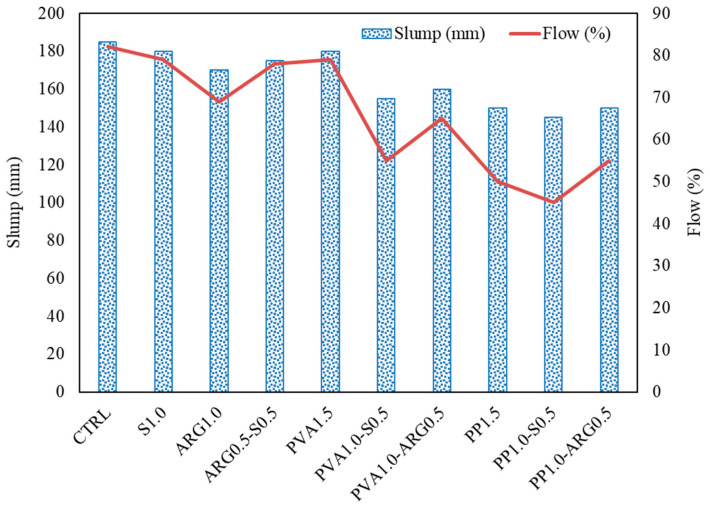
Slump and flow of fresh geopolymer mortars with mono and hybrid fibres.

Workability is a key parameter for fibre-reinforced composites since adequate workability is essential for proper placement, compaction and finishing of the material. Workability is specifically important for geopolymers due to the use of alkaline solutions instead of water which affects viscosity of the mix. Also, workability of geopolymers can decrease fast depending on the rate of reaction of the aluminosilicate precursors with the alkaline liquid. Photographs of the spread of typical fresh geopolymer mortar mixtures in the flow test are shown in Figure 2. The slump and flow values of the control mixture and the mixtures containing different fibres are presented in Figure 3.

It can be seen from Figure 2 that the control mixture was plastic in nature with a high flowability after mixing. However, the consistency of the mixture became stiffer with the addition of fibres. It can be noted that the geopolymer mixtures used a liquid to solid ratio of 0.6 and fibre volume of 1 or 1.5%. It can be seen from Figure 3 that the spread of the mixtures in the flow test showed similar trends with that of slump. 

Flow of the geopolymer mortar without any fibre was 82% and those of the mortars using 1% steel fibre or 1.5% PVA fibre were 79%, which was marginally less than that of the control mixture. Flow of the mortar with 1% ARG fibre was 69%. The flow further decreased to 50% with the use of 1.5% PP fibres resulting in a relatively stiff mixture. Generally, the workability of fibre-reinforced geopolymer mortar is affected by the surface area, length and surface morphology of the fiber. The reason for a large reduction of flow by the PP fibres is that these fibres were dispersed into individual filaments more than the ARG and PVA fibres. As the bundled fibres are filamentised, the surface area of the fibres is significantly increased which increases the liquid demand. Thus, the filamentised fibres used more liquid than the partially bundled fibres and the workability of mortar decreased with the increase of the filamentisation of bundled fibres. Generally, more filamentisation was observed in the bundled PP fibres than the PVA and ARG bundled fibres in the geopolymer mortar mixtures. For this reason, the workability of geopolymer mortar using PP fibres was less than those of mortars using PVA and ARG fibres, as indicated by the slump and flow values. Regarding workability of the mortars with hybrid fibres, it can be seen from Figure 3 that flow increased when a part of the ARG, PVA or PP fibre was replaced by steel fibre. This is attributed to the smaller surface area of steel fibres than the other types of fibres. Hybridisation of any type of fibre with PP fibres was seen to result in reduced workability due to its higher dispersion into individual filaments that increased the total surface area of the dispersed fibres.

### 3.2. Compressive Strength

**Figure 4:**
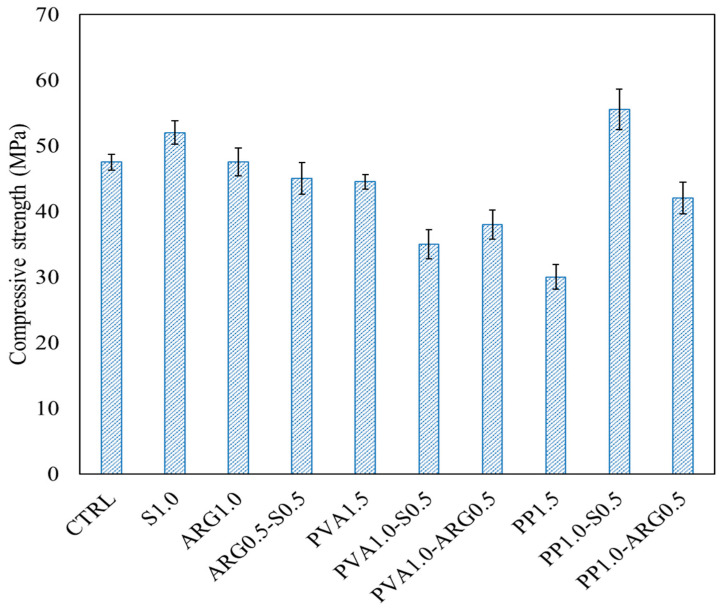
Compressive strengths of geopolymer mortars with mono and hybrid fibres.

**Figure 5:**
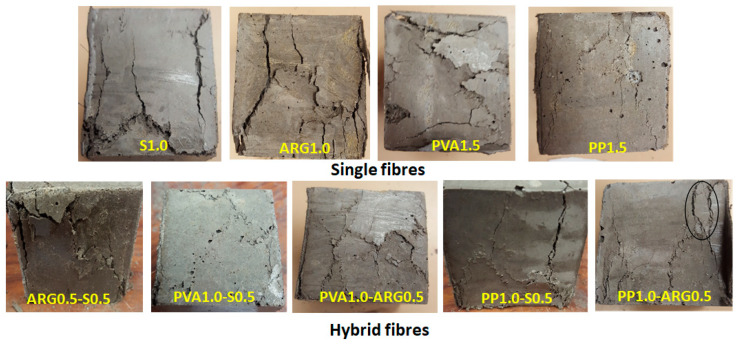
Failure patterns of the FRGM cubes in compressive strength tests.

The mean compressive strengths of the control mortar and the mortars with different types of fibres are shown in Figure 4, with the error bars at one standard deviation. The typical failure patterns of the cube specimens under compressive load are also shown in Figure 5. The mean compressive strength of the control geopolymer mortar was 48 MPa. It can be seen that the geopolymer mortar with 1% steel fibre had a mean compressive strength of 52 MPa, which is 8% higher than that of the control mortar. The increase of compressive strength is attributed to the high tensile strength and elastic modulus of the steel fibres. The mortar specimens with 1% ARG fibres had a compressive strength of 48 MPa, which was the same as that of the control mixture. Thus, 1% ARG fibres maintained the same compressive strength of the control geopolymer mortar and 1% steel fibres slightly increased the compressive strength.

The mortar with 1.5% PVA fibres had a compressive strength of 45 MPa, which is 7% less than that of the control mortar. The mortar with 1.5% PP fibres had a mean compressive strength of 30 MPa, which is 38% lower than that of the control mortar. The decrease of compressive strength of the specimens with PP fibres is attributed to the presence of voids and pores that can be found in the mortars containing large number of fibres. Since the PP fibres were dispersed very well in geopolymer mortar after filamentisation, failure of these specimens was characterised by many thin cracks. On the other hand, failures of the specimens with ARG and PVA fibres were characterised by fewer and wider cracks due to less filamentisation of these fibres as compared to the PP fibres. The significant decrease of compressive strength of the specimens with PP fibres is also attributed to the very low tensile strength and elastic modulus of the PP fibres as compared to those of the other fibres, as shown in Table 2. 

The ARG0.5-S0.5 hybrid FRGM specimens had a compressive strength of 45 MPa, which is close to that of the control mortar. The PP1-S0.5 hybrid FRGM showed a compressive strength of 55 MPa, which is 14% higher than that of the control mortar. However, compressive strength of the PP1-ARG0.5 hybrid FRGM was 42 MPa. On the other hand, combinations of 1% PVA fibres with 0.5% steel fibres and 0.5% ARG fibres resulted in compressive strengths of 35 MPa and 38 MPa, respectively. These were significant reductions of compressive strength than that of the control mortar. Therefore, the combination of 1% PP fibres with 0.5% steel fibres resulted in the highest compressive strength among all the mixtures. The reason for the highest compressive strength of this combination of fibres is that while the well-dispersed PP fibres could delay the initiation of micro cracks in the composite, the higher modulus steel fibres were able to bridge the major fractured substrates better than the other fibres.

### 3.3. Flexural Behaviour

Flexural strengths were calculated corresponding to the first crack and the peak value of the load. The toughness parameters were calculated from the area under the load-deflection diagram. According to ASTM C1609 [22], three toughness indices, namely I5, I10 and I20, are defined as the ratio of areas at 3, 5.5, and 10.5 times of the first crack deflection to the area up to the first crack deflection, respectively. In addition, the residual strength factors, namely R5,10 and R10,20, were calculated using the toughness indices as 20 × (I10–I5) and 10 × (I20–I10), respectively. The residual strength factor represents the level of strength retained by the specimen for the specified range of deflections after occurrence of the first crack as a percentage of the first crack strength. Thus, the factor R5, 10 represents the average percentage of the first crack strength retained in the post-crack range of deflections between 3 times and 5.5 times of the first crack deflections. Similarly, the factor R10,20 represents the average percentage of first crack strength retained in the range of post-crack deflections of 5.5 times and 10.5 times of the first crack deflection. A residual strength factor of 100 or more is considered to show deflection hardening behaviour and a value of zero is assigned to plain concrete. The residual strength factors were used to compare the post-cracking performances of the different FRGM specimens, with a higher value indicating a better performance. 

#### 3.3.1. Flexural Strength and Toughness of FRGM with Mono Fibres 

**Figure 6:**
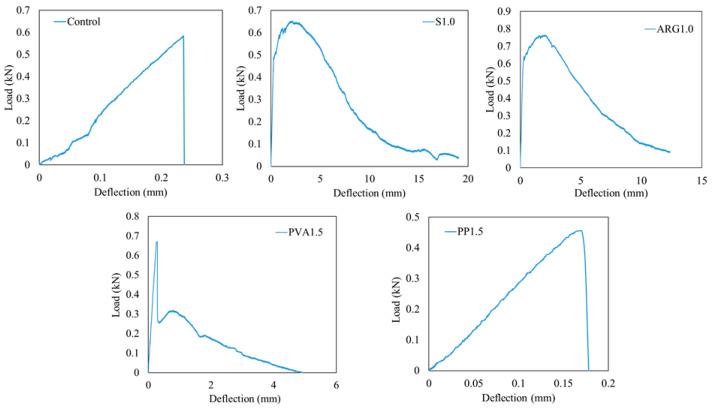
Load versus mid-span deflection of specimens using mono fibres.

**Figure 7:**
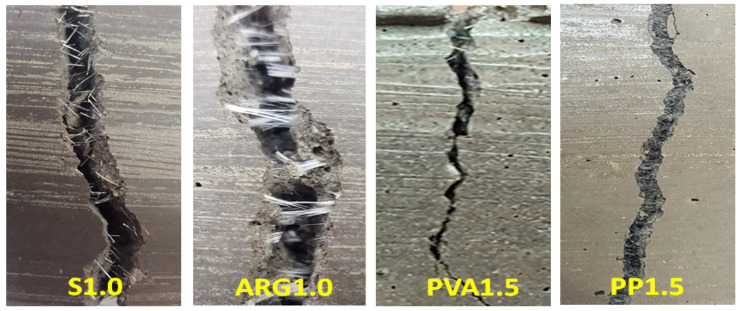
Failure patterns in flexural testing of specimens with mono fibres.

The load versus deflection diagrams and images of failure at the tensile face of the FRGM specimens with mono fibres are presented in Figure 6 and Figure 7, respectively. The flexural strengths and toughness parameters are given in Table 4. As expected, the control mortar specimens (without fibre) failed suddenly after showing the first crack at the peak load, which can be observed in Figure 6.

**Figure 8:**
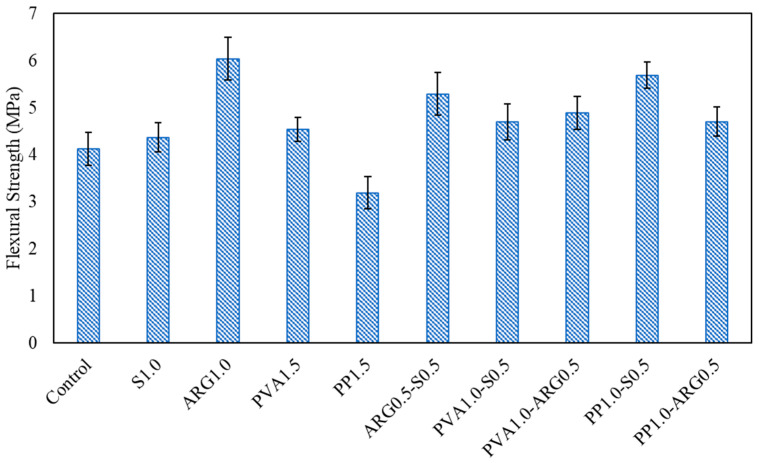
Flexural strengths of geopolymer mortars with mono and hybrid fibres.

It can be seen from Table 4 that the load bearing capacity of steel fibres were activated after the first crack, where the flexural strength was 35.7% higher than the first crack strength. The first crack strength and flexural strengths exhibited a 22.1% decrease and 5.76% increase, respectively, as compared to the control specimen. Similarly, the ARG1.0 FRGM specimens exhibited a 46.1% increase of the first crack strength as compared to that of the control specimens. It is also evident that there was relatively low filamentisation of the ARG fibres in the geopolymer mortar mixtures. The PVA1.5 specimens showed a 9.6% increase of the first crack strength and flexural strength as compared to those of the control specimens. The mean values of flexural strengths of the FRGM specimens are plotted in Figure 8 with the error bars at one standard deviation. It can be seen that the PP1.5 FRGM specimens had a flexural strength of 3.18 MPa, which is 22.8% less than that of the control specimens. This is attributed to the low workability and very low elastic modulus of the PP fibres. The samples exhibited high elongation of fibres with the propagation of cracks, as shown in Figure 7.

The ASTM C1609 [22] toughness indices, namely I5, I10 and I20, were calculated using the area under the load-deflection curves. The residual strength factors R5,10 and R10,20 were calculated using the toughness indices as per the standard. The average I5, I10 and I20 indices of the steel FRGM specimens were found to be 5.6, 12.0 and 25.4, respectively. According to ASTM C1609 [22], a composite is considered to have deflection hardening property when I5 > 5, I10 > 10 and I20 >2 0. Therefore, the S1.0 FRGM specimens are considered to have shown deflection hardening behaviour. The residual strength factors R5,10 and R10,20 of these specimens were 127 and 134, respectively, which were highest among all the FRGMs using mono fibres. The final failure of the S1.0 FRGM specimens occurred by pull out of the fibres. No fracture of fibres was observed in these specimens, as shown in Figure 7. Multiple cracks appeared before the final fracture of the specimens through a dominant crack.

Failure of the ARG1.0 and PVA1.5 specimens were associated with pull out and fracture of fibres, as shown in Figure 7. The toughness indices of the ARG1.0 specimens were less than the specified values for deflection hardening behaviour. Multiple cracks were also observed in these specimens before the final failure. As shown in Figure 6, the PVA1.5 specimens showed a large drop of the load immediately after reaching the first peak and then a small increase to a second peak load. A clear softening load-deflection behaviour with relatively low end-point deflections was shown by these specimens as compared to the S1.0 and ARG1.0 specimens. The toughness indices of these specimens were less than those specified for deflection hardening behaviour with relatively low residual strength factors. 

As shown by the load-deflection curve of Figure 6, the PP1.5 FRGM specimens did not show any toughness and residual strength. The flexural behaviour of the PP1.5 FRGM specimens were similar to those shown by the control specimens due to the very low elastic modulus of the PP fibres. Therefore, the toughness indices of these specimens were 1.0 and the residual strength factors were zero.

#### 3.3.2. Flexural Strength and Toughness of Hybrid Fibre-reinforced Specimens

**Figure 9:**
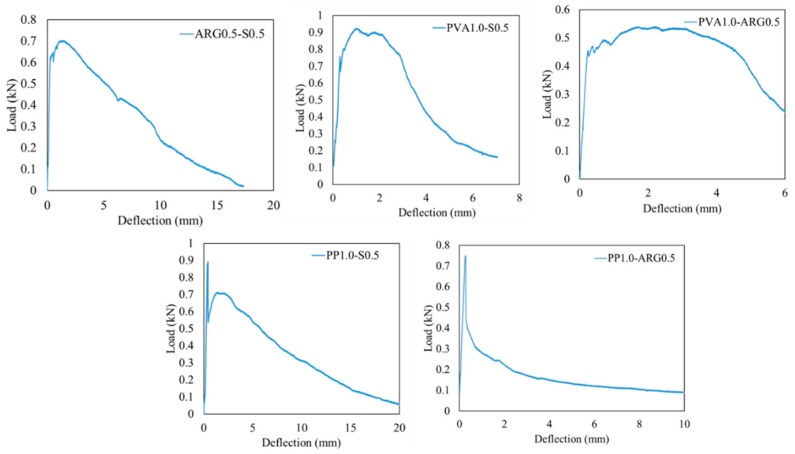
Load versus mid-span deflection of specimens using hybrid fibres.

**Figure 10:**
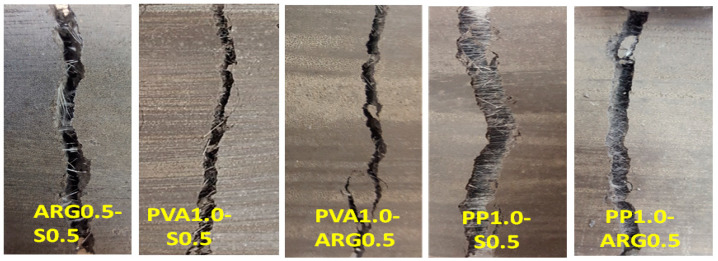
Failure patterns in flexural test of FRGM specimens with hybrid fibres.

The representative load versus deflection diagrams and the fracture plane at failure of the hybrid FRGM specimens are shown in Figure 9 and Figure 10, respectively. The flexural strengths are plotted in Figure 8 and the toughness parameters are given in Table 5.

The ARG0.5-S0.5 FRGM specimens exhibited a mean first crack strength of 4.56 MPa and mean flexural strength of 5.29 MPa. Thus, the flexural strength increased by 21% as compared to that of the S1.0 FRGM. As shown in Figure 9, the load gradually dropped after reaching the peak value until the final failure at a large end-point deflection. These specimens showed the highest deflection at peak load among all the FRGM specimens. It can be seen from the toughness indices in Table 5 that these specimens showed marginal deflection hardening according to ASTM C1609 [22]. The residual strength factors R5,10 and R10,20 were greater than 110, indicating the high toughness of these specimens.

The PVA1.0-S0.5 specimens showed a similar flexural strength as that of the PVA1.5 specimens. These specimens also showed marginal deflection hardening behaviour as indicated by the toughness indices in Table 5. This is due to the enhanced post-crack load deflection behaviour, as shown in Figure 9. The residual strength factors of these specimens were similar to those of the ARG0.5-S0.5 specimens. The PVA1.0-ARG0.5 specimens showed similar flexural strength with lower toughness indices and residual strength factors as compared to those of the ARG0.5-S0.5 specimens.

As shown in Figure 8, the PP1.0-S0.5 specimens showed a flexural strength of 5.69 MPa, which was the highest flexural strength among all the FRGM specimens. This is consistent with the fact that these specimens also showed the highest compressive strength, as shown in Figure 4. As shown in Figure 9, there was a sudden drop in the load after reaching the peak value which is attributed to the very low stiffness of the PP fibres. The significant elongation of the low modulus PP fibres in the crack can be seen in Figure 10. However, the load then increased to a second peak before a gradual decrease until final failure with a large end-point deflection. The increase of the load to a second peak is attributed to the effect of the high modulus steel fibres after occurrence of the crack at the first peak load. The toughness indices, I5, I10 and I20 were 3.94, 7.8 and 15.4, respectively. The residual strength factors were also lower than those of the PVA1.0-S0.5 specimens. Thus, these specimens did not show deflection hardening behaviour in terms of the toughness indices though there was a large end-point deflection at failure.

It can be noted that though the PVA and ARG mono fibres exhibited low residual strength factors (Table 4), their hybridisation with steel fibres exhibited enhancement of the R10,20 factor by 78 and 82%, respectively.

The PP1.0-ARG0.5 specimens showed a flexural strength of 4.7 MPa, which is same as that of the PVA1.0-S0.5 specimens. However, there was a large and sudden drop of the load after reaching the peak value. The load then dropped gradually until failure of the specimen, as shown in Figure 9. The effect of the hybridisation of two fibres of low elastic modulus is reflected in the very low values of toughness indices and residual strength factors of these specimens, as shown in Table 5. The specimens showed clear deflection softening behaviour with the lowest toughness indices and residual strength factors among all the hybrid FRGM specimens.

## 4. Conclusions

The effects of steel, ARG, PVA and PP short fibres on the workability, compressive strength, flexural strength and toughness parameters of fly ash geopolymer mortar were studied. The fibres were used in mono and hybrid combinations at a rate of 1 or 1.5% volume of the mortar. The geopolymer mortar mixtures consisted of a high sand to fly ash mass ratio of 2.75 and an alkaline liquid to fly ash mass ratio of 0.6. The following conclusions are drawn from the experimental results:

- Flow of the freshly mixed control geopolymer mortar (without fibre) was 82%, which marginally decreased by the use of 1% steel or 1.5% PVA fibres. The flow of geopolymer mortar further decreased to 69% with 1% ARG fibres and to 50% with 1.5% PP fibres. Thus, workability generally decreased with the use of fibres.
- Compressive strengths of FRGMs S1.0, ARG1.0 and PVA1.5 were similar to that of the control mortar which was 48 MPa. A 14% increase in compressive strength was found in the mortar PP1.0-S0.5. However, a 30% decrease of compressive strength than that of the control mortar was observed in FRGM PP1.5. The decrease of strength is attributed to the entrapped air voids in these specimens due to the low workability caused by high filamentisation of the 1.5% volume of PP fibres. Similarly, decreases of compressive strength were observed in the FRGMs PVA1.0-ARG0.5 and PVA1.0-S0.5 against the control mortar.
- Generally multiple cracks were initiated in the tensile face of the FRGM specimens before the final failure through a dominant crack. The flexural failures of the FRGM specimens were characterised by pull out of steel fibres, significant elongation of the PP fibres and full out and fracture of the ARG and PVA fibres. Flexural strength of FRGM increased with the use of mono or hybrid fibres at 1 or 1.5% dosage except for the mortar PP1.5, which showed a decrease of flexural strength. The reason for the decrease of flexural strength is attributed to the low workability and low elastic modulus of the PP fibres.
- Deflection hardening behaviour in terms of the ASTM C1609 [22] toughness indices I5, I10 and I20 were exhibited by the FRGMs S1.0, ARG0.5-S0.5 and PVA1.0-S0.5 specimens. These specimens also showed residual strength factors more than 100. The toughness indices and residual strength factors of the FRGMs using other mono or hybrid fibres were relatively low. The post-cracking load-deflection curve of the PP1.5 specimens was similar to that of the control specimen with zero residual strength factors due to very low elastic modulus of the PP fibres. However, hybridisation of the PP fibres with high modulus steel fibres increased both flexural strength and the toughness indices of FRGM.
- In summary, PP fibres generally showed more decrease of workability either in mono or hybrid form due to its higher dispersion to individual filaments compared to the bundled ARG and PVA fibres. Multiple cracking and deflection hardening behaviours could be achieved in fly ash geopolymer mortars of high sand to binder ratio with 1% steel mono fibre and 0.5% steel with 0.5% ARG or 1% PVA hybrid fibres.

## Figures and Tables

**Table 1:** Geopolymer mortar mixture proportions (1 m^3^).

Materials	Fine Sand	Fly Ash	Na_2_SiO_3_ Solution	NaOH Solution	Plasticiser
Mass (kg)	1367	497	213	85	6

**Table 2:** Physical and mechanical properties of fibres.

Fibre Type	Length (mm)	Diameter (μm)	Aspect Ratio	Tensile Strength (MPa)	Elastic Modulus (GPa)	Density (g/cm^3^)
Steel (S)	10	120	83	2500	200	7.8
AR glass (ARG)	13	18	720	1500	74	2.8
Polyvinyl alcohol (PVA)	8	38	210	1600	40	1.3
Polypropylene (PP)	6	32	188	450	3.5	0.91

**Table 3:** Mix designations and volume percentage of different fibres.

Mix Code	Fibre Combinations and Volume Percentage of Fibres	Total Fibre Content
S1.0	Steel (S)	1.0%
ARG1.0	Alkali resistant glass (ARG)	1.0%
ARG0.5-S0.5	Steel (0.5%) + ARG (0.5%)	1.0%
PVA1.5	Polyvinyl alcohol (PVA)	1.5%
PVA1.0-S0.5	PVA (1%) + Steel (0.5%)	1.5%
PVA1.0-ARG0.5	PVA (1%) + ARG (0.5%)	1.5%
PP1.5	Polypropylene (PP)	1.5%
PP1.0-S0.5	PP (1%) + Steel (0.5%)	1.5%
PP1.0-ARG0.5	Polypropylene (1%) + ARG (0.5%)	1.5%

**Table 4:** Mean flexural strength and toughness of FRGM specimens with mono fibres.

Parameter		Control	S1.0	ARG1.0	PVA1.5	PP1.5
First crack strength (MPa)		4.13	3.22	6.03	4.53	3.19
Flexural strength (MPa)		4.13	4.37	6.04	4.54	3.19
Toughness indices	I_5_	1	5.60	3.86	2.80	1
	I_10_	1	11.98	6.13	4.96	1
	I_20_	1	25.39	8.08	7.61	1
Residual strength factors	R_5,10_	0	127.54	45.28	43.16	0
	R_10,20_	0	134.08	19.56	26.57	0

**Table 5:** Mean flexural strength and toughness parameters of FRGM with hybrid fibres.

Parameters		ARG0.5-S0.5	PVA1.0-S0.5	PVA1.0-ARG0.5	PP1.0-S0.5	PP1.0-ARG0.5
First crack strength (MPa)		4.56	4.02	4.89	5.67	4.7
Flexural strength (MPa)		5.29	4.7	4.89	5.69	4.7
Standard deviation flexural strength		0.45	0.38	0.35	0.28	0.31
Toughness indices	I_5_	5.06	4.95	4.28	3.94	2.74
	I_10_	10.61	10.38	8.6	7.8	4.37
	I_20_	21.7	21.35	16.52	15.4	6.65
Residual strength factors	R_5,10_	111	108.44	86.47	77.14	32.66
	R_10,20_	110.98	109.7	79.21	76.04	22.74

## Data Availability

The data presented in this study are available on request from the corresponding author.
